# Physiological plasticity related to zonation affects *hsp70* expression in the reef-building coral *Pocillopora verrucosa*

**DOI:** 10.1371/journal.pone.0171456

**Published:** 2017-02-15

**Authors:** Davide Poli, Elena Fabbri, Stefano Goffredo, Valentina Airi, Silvia Franzellitti

**Affiliations:** 1 Department of Biological, Geological and Environmental Sciences, University of Bologna, via S. Alberto, Ravenna, Italy; 2 Interdepartment Centre for Environmental Sciences Research, University of Bologna, via S. Alberto, Ravenna, Italy; 3 Marine Science Group, Department of Biological, Geological and Environmental Sciences, University of Bologna, Via F. Selmi 3, Bologna, Italy; Boston University Medical School, UNITED STATES

## Abstract

This study investigates for the first time the transcriptional regulation of a stress-inducible 70-kDa heat shock protein (*hsp70*) in the scleractinian coral *Pocillopora verrucosa* sampled at three locations and two depths (3 m and 12 m) in Bangka Island waters (North Sulawesi, Indonesia). Percentage of coral cover indicated reduced habitat suitability with depth and at the Tanjung Husi (TA) site, which also displayed relatively higher seawater temperatures. Expression of the *P*. *verrucosa hsp70* transcript evaluated under field conditions followed a depth-related profile, with relatively higher expression levels in 3-m collected nubbins compared to the 12-m ones. Expression levels of metabolism-related transcripts ATP synthase and NADH dehydrogenase indicated metabolic activation of nubbins to cope with habitat conditions of the TA site at 3 m. After a 14-day acclimatization to common and fixed temperature conditions in the laboratory, corals were subjected for 7 days to an altered thermal regime, where temperature was elevated at 31°C during the light phase and returned to 28°C during the dark phase. Nubbins collected at 12 m were relatively more sensitive to thermal stress, as they significantly over-expressed the selected transcripts. Corals collected at 3 m appeared more resilient, as they showed unaffected mRNA expressions. The results indicated that local habitat conditions may influence transcription of stress-related genes in *P*. *verrucosa*. Corals exhibiting higher basal *hsp70* levels may display enhanced tolerance towards environmental stressors.

## Introduction

Anthropogenic stressors, such as climate changes, are driving relevant shifts in the abiotic features of marine ecosystems. Such changes are occurring much faster than adaptive capacity of marine organisms [1], so that greater concern in environmental physiology is whether extant marine species possess the potential to adapt to these ongoing environmental challenges.

Reef-building corals, the foundation of tropical coastal marine ecosystems, occupy a narrow range of oceanographic features making them exceptionally vulnerable to climate changes [2,3]. It is estimated that 30% of reefs are already severely damaged, and close to 60% may be lost by 2030 [2,4]. Drivers of climate change, including alterations in ocean temperature, oxygen availability, salinity, pCO_2_, and pH, may considerably affect physiology and adaptive potential of reef organisms over extended geographic and time scales [5]. Increases in seawater temperature are among those factors related to mass mortality of corals due to bleaching [6,7], also acting synergistically with other threats, including pathogen infections [8], changes of nutrient supplies [9], or exposure to pollutants [10]. Field data and experimental exposures under controlled conditions showed that ocean acidification levels as high as those expected by 2100 [11] may compromise calcification efficiency and change coral morphology, leading to a more porous and potentially fragile phenotype [12–14]. Further physiological processes, including reproduction, metabolic rates, and stress-response may also be affected [6,7]. Recent laboratory studies [3] and the discovery of natural populations resistant to extreme temperature events [15] or to naturally occurring hypercapnia [16] have shed new light on coral acclimatization and adaptive capabilities. These evidence lead to hypothesize that in such long-lived organisms physiological acclimatization rather than genetic adaptation will play the leading role in their response to climate changes [3].

Environmental acclimatization describes the process of tuning physiology of organisms within their lifetime allowing them to cope with varying environments, and it is also referred to as phenotypic plasticity [17]. Investigations of the molecular mechanisms underlying acclimatization in corals have become increasingly employed as they may aid in assessing, predicting and managing the impacts of ocean changes [18,19]. Coordinated modulation of gene expression represents one of the most rapid and versatile reaction available to organisms experiencing environmental stress [20]. Monitoring changes in mRNA expression profiles may provide early-warning insights into physiological mechanisms governing stress responses [21], forecasting possible climate effects on ecosystems at species and community levels [22].

The induction of heat shock proteins (Hsps; [23]) is one of the most conserved and ubiquitous physiological mechanism associated with environmental acclimatization [24]. Hsps are molecular chaperones that regulate protein structure and function under physiological conditions [25]. They are addressed to as key components of the “minimal” or “core” cellular stress response, i.e. a suite of proteins and molecular processes highly conserved throughout metazoan, responding in a coordinated fashion to a multitude of exogenous stimuli [24]. In response to proteotoxic stressors, Hsp expression is promptly induced to preserve protein structure and functions, and promote cellular repair processes and tolerance to adverse conditions [26]. Hsps are classified into major families according to their molecular weight, e.g., Hsp100, Hsp90, Hsp70, Hsp27, etc. [23]. Enhanced thermotolerance in many marine organisms, including intertidal invertebrates as limpets, mussels, oysters, sea cucumbers, and amphipods, has been linked to higher physiological expression of different stress-inducible members of the 70-kDa Hsp family (Hsp70) [27–31].

This study investigates transcriptional response of a stress-inducible Hsp70 in the important reef-building scleractinian coral of the Red Sea and Pacific Ocean, *Pocillopora verrucosa* (Ellis and Solander, 1786). This zooxanthellate species is widely distributed in shallow-water high-light environments from fringing reefs to exposed reef fronts [32]. *P*. *verrucosa* nubbins were sampled at different sites and depths along the Eastern side of the Bangka Island (North Sulawesi, Indonesia), within the Coral Triangle area. This area of the Pacific Ocean hosts about the 76% of the World’s total zooxanthellate coral species (about 605 species) [33]. Individual reefs in this area have up to 280 species ha^-1^, four times higher than total zooxanthellate scleractinian species richness of the entire Atlantic Ocean [34], making the Coral Triangle a hot spot of biodiversity [35], and ideal habitats for coral reefs [36]. In light of predicted impacts of climate changes on such ideal habitats [33,36,37], the ability of corals within the region to physiologically adapt from their current physico-chemical habitat to another is a critical question that deserves to be addressed. By combining field samplings and laboratory experiments, the study attempts to assess transcriptional regulation of the Hsp70 response in this coral species under a short-term thermal stress exposure and to discuss possible influences of life history traits and local environment constraints. Furthermore, expression of transcripts encoding ATP synthase (*ATPs*) and NADH dehydrogenase (*ndh*) was investigated to account for stress effects on host cnidarian oxidative metabolism and regulation of symbiosis [38,39]. Initiation of the response to bleaching is believed to stem from the decoupling of photosynthesis which results in damage of the photosystem II apparatus and in a subsequent production of reactive oxygen species (ROS) [38]. The corresponding membrane and protein damage from excess ROS production in both the coral host and the symbiont lead inevitably to a breakdown in carbon fixation, ATP and NADH production (Ref. [38] and reference therein). Changes of *ATPs* and *ndh* expressions under hyperthermic stress have been related with an altered capacity of the host to mitigate oxyradical generation and to maintain integrity of cell components and of energy supplies [40], leading to initiation of pro-apoptotic pathways in irreparable cells, or at the onset of delayed protective responses (involving over-expression of anti-apoptotic genes) in surviving cells [41,42].

## Methods

### Ethic statement

All experimental procedures were approved by the Ethical and Scientific Committee of the University of Bologna and were carried out in accordance with Indonesian legislation regarding the protection of animals used for experimental and other scientific purposes. The sampled reefs do not fall under any legislative protection or special designation as a marine/environmental protected area. *Pocillopora verrucosa* is listed as at “Least Concern” under the IUCN Red List [32], so that it is not subjected to any special protection.

### Coral sampling and experimental design

**Fig 1:**
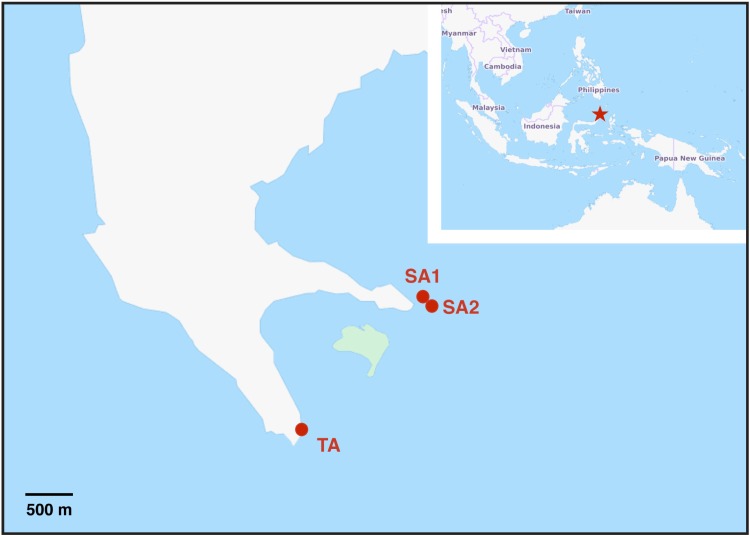
Map of the sampling sites at Bangka Island (North Sulawesi, Indonesia). SA1: Sahaoung 1; SA2: Sahaoung 2; TA: Tanjung Husi. Maps were generated using the OpenStreetMap dabase (http://www.openstreetmap.org/copyright).

Experiments were performed at the CoralEye Reef Outpost at the Bangka Island (North Sulawesi, Indonesia) in July-August 2014 (Fig 1). *Pocillopora verrucosa* nubbins were collected by SCUBA diving at three different sampling locations in the South-East side of the Bangka Island (Fig 1). SA1 and SA2 are banks located about 400 m off the coast, while TA is an integral part of the coastal reef. Direct field observations indicated that the prevalent current at the TA site flows parallel to the coast and are mainly driven by tidal changes, while a more complex circulation occurred around SA1 and SA2 sites, which are also more exposed to the open seawaters. *In-situ* measurements of water parameters at the time of coral collection for each site were performed with a multi-parametric probe (ADWA AD12) and densitometer (Milwaukee MR100 ATC). Data are reported in Table 1. Satellite-derived data for physical parameters across a 3- to 4- months period spanning the sampling time point are reported in the S1 Fig.

**Table 1:** *In-situ* measurements of water parameters upon coral collection from the sampling sites.

	Sahoung 1		Sahoung 2		Tanjung Husi	
	(SA1)		(SA2)		(TA)	
GPS position	1°44’38.026” N		1°44’39.956” N		1°44’14.94” N	
	125° 9’49.845” E		125° 9’43.298” E		125° 9’ 16.162” E	
Orientation	N-E		N-E		N-E	
Depth (m)	3	12	3	12	3	12
Temperature (°C)	27.7	27.0	27.7	27.0	29.4	28.0
pH	8.81	8.57	8.68	8.85	8.77	8.72
Salinity (psu)	34	35	35	34	35	35

Coral cover at each site was estimated by visual census with a Line Intercept Transect method according to Bianchi et al. [43]. Visual census was performed along 6 linear 10-m horizontal transects (indicated by a line) placed at 3 m and 12 m. The divers counted the number of colonies visible in a distinct 1 m wide visual field on each side of the line. The 12-m depth was chosen because water temperature became stable below 10 m.

**Fig 2:**
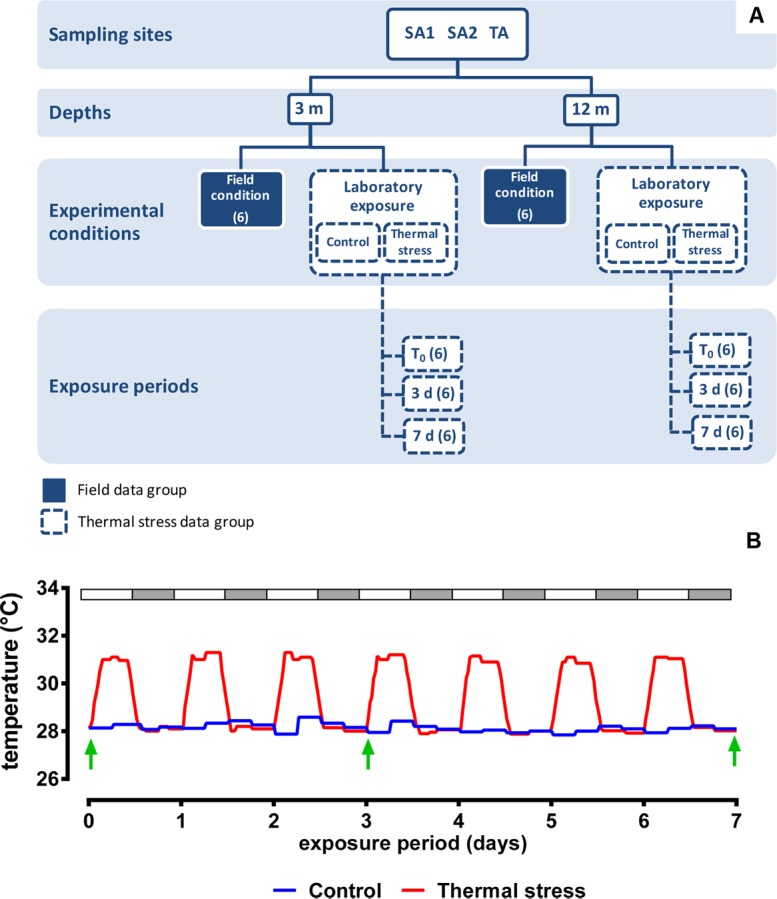
The experimental setup (see Supporting information S1 File for details). **(A)** Schematic flowchart of the experimental design. Replicates for each condition are given in brackets. T_0_: time zero of the thermal stress exposure; 3d: 3-days sampling point; 7d: 7-days sampling point. **(B)** Thermal stress profile simulated in the experiment. Open and grey-filled bars on top of the graph indicate light/dark daily cycles (10L:14D). Green arrows indicate the sampling points.

A detailed description of the experimental setup is reported in Supporting Information S1 File and summarized in Fig 2. Thermal stress experiment were carried out using a “nocturnal recovery” experimental profile (Fig 2B) [44], in which 6 randomly selected aquaria were maintained at a constant temperature of 28°C ± 0.5°C, while further 6 randomly selected aquaria were assigned to the thermal stress group, in which water temperature was elevated at 31°C ± 0.5°C during the light phase, while reduced to 28°C ± 0.5°C during the dark phase (Fig 2B; Supporting Information S1 File). Samplings from both the control and thermal-stress groups were performed at the same time of the day at which corals were collected from the field and after 3 and 7 days of treatment exposure (Fig 2B). Samples were immediately preserved in the RNAlater® solution (Sigma Aldrich, Milan, Italy) and stored at– 20°C until analysed.

### RNA extraction and cDNA preparation

A small piece of coral branch (about 1 cm^2^) was mechanically homogenized in a suitable volume of the Tri-Reagent (Sigma Aldrich, Milan, Italy) according to Barshis et al. [45]. Total RNA was extracted using the DirectZol kit (Zymo Research, Freiburg, Germany) according to the manufacture’s protocol. DNAase I treatment was performed within the RNA extraction procedures with the DirectZol kit according to the manufacturers’ instructions (Zymo Research, Freiburg, Germany). RNA concentration and quality were verified through the Qubit RNA assay (Life Technologies, Milan, Italy) and electrophoresis using a 1.2% agarose gel under denaturing conditions. RNA integrity was evaluated based on clear 28S and 18S ribosomal RNA bands in the electrophoresis. First strand cDNA for each sample was synthesized from 600 ng total RNA using the iScript supermix following the manufacturer’s protocol (Biorad Laboratories, Milan, Italy).

### qPCR assays

Transcriptional analyses were performed by quantitative Real Time Polymerase Chain Reaction (qPCR) assays and using a protocol for the absolute quantification of the target transcripts. This approach was selected to overcome the impossibility in identifying stably expressed reference transcripts, which is a demanding issue in relative quantification qPCR studies [46], due to the lack of extensive genomic/transcriptomic information on *P*. *verrucosa*.

Target-specific primer pairs (*hsp70*, *ATPs*, *ndh*) were designed with the Primer Express software (Life Technologies, Milan, Italy) using nucleotide sequences retrieved from the GenBank database (https://www.ncbi.nlm.nih.gov/genbank/) for *P*. *verrucosa* (Table 2; see S2 Fig and S1 Table for Hsp70 protein sequence classification). qPCR standards for each target transcript were prepared by serial dilution of the linearized plasmid DNAs containing the specific transcripts to obtain a standard curve of C_T_ values *vs* the logarithmic DNA amount. R^2^ values for all standard curves were > 0.99. Absolute mRNA abundance was calculated from the standard curves and plotted as copy number ng^-1^ RNA (mean ± s.e.m.). Reactions were performed in a final volume of 10 μL containing 5 μL iTaq Universal SYBR Green Supermix with ROX (Bio-Rad Laboratories, Milan, Italy), 2 μL diluted cDNA or plasmid DNA, and 0.2 μM specific primers (Table 2). A control lacking the DNA template (no-template) and a minus-reverse transcriptase (no-RT) control were included in the qPCR analysis to ensure the specificity of the amplification.

**Table 2:** Primers and qPCR parameters.

Gene	Primer sequence	Amplicon size	Tm	PCR efficiency^a^	GenBank Accession Number
	(5’→3’)	(bp)	(°C)	(%)	
***ATPs***	CGGTCCATCCTTGAGCTTATT	128	59.5	Samples: 102.6 ± 1.9	JX985612
	GGGTACAAGTGAATCAAGAGTCT		60.9	Standards: 104.7 ± 0.7	
***ndh***	ATTCGGGCTCGTTTAGCGAT	109	59	Samples: 98.0 ± 0.8	KF583919
	CATCTCACCCCTCCACGAAG		62	Standards: 99.0 ± 0.7	
***hsp70***	TCGCGTACGGTTTGGAGAAA	132	58.4	Samples: 104.2 ± 1.4	JX624896
	CAGCTGTGGAGAGAACCTGG		62	Standards: 103.1 ± 1.6	

^a^ PCR efficiencies for samples and standards are reported as mean ± s.d.

Tm: melting temperature.

Technical replicates have been performed both within each run/plate (samples in duplicate, qPCR standards in triplicate) and between different runs/plates (standards and samples replicated on different plates). Equal loadings within each qPCR reaction were ensured by checking the amounts of each standard and cDNA sample using the Qubit system with Qubit® dsDNA HS (High Sensitivity) assay kit (Thermo Scientific, Milan, Italy).

Amplification was detected with a StepOne real time PCR system (Life Technologies, Milan, Italy) using a standard “fast mode” thermal protocol. For each target mRNA, melting curves, gel pictures and sequences of PCR products were analyzed to verify the specificity of the amplified products and the absence of artifacts. About 20 positive clones for each PCR product were sequenced, all of which resulted identical to the nucleotide sequences of *P*. *verrucosa* originally employed for designing the selected primer pairs. Although some contaminations due to *Symbiodinium* transcript amplifications cannot be excluded [47], we are confident that the employed qPCR protocols do mostly account for changes in mRNA expression profiles of *P*. *verrucosa*.

The amplification efficiency of each primer pair was calculated using a dilution series of samples cDNAs or of the linearized plasmid DNAs for the qPCR standards (Table 2). PCR efficiencies of the standards were not significantly different from those of the samples (p > 0.05; Mann-Whitney U-test). A standard curve was included in any PCR run. Consistency of technical replicates within each run was checked as a default operation by the StepOne software (standard deviations between replicates < 0.5). Consistency of data between different runs was assured by analyzing reproducibility of C_T_ values obtained from qPCR standards or randomly selected samples from any experimental condition (P > 0.05 according to the Mann-Whytney U-test). Consistency of parameters of standard curves belonging to different runs was also assessed (P > 0.05 according to the Mann-Whytney U-test).

### Statistical analysis

Statistical analyses were performed by permutation multivariate analysis of variance (PERMANOVA) using the PERMANOVA+ add-on in PRIMER v6 (PRIMER-E Ltd, UK). Percentages of *P*. *verrucosa* cover were used to calculate a similarity matrix based on the Bray-Curtis similarity measure (999 permutations). Log-transformed copy number variations of the target transcripts from field data group and thermal stress data group, respectively, were used to calculate similarity matrices based on the Euclidean distance (999 permutations). “Sampling site” and “depth” were selected as fixed factors in coral cover data as well as the field data group for mRNA expressions in order to determine differences between corals sampled at different locations and depths. For the thermal stress data group, factors considered were “sampling site”, “depth”, “exposure time”, and “treatment”. Pseudo-F values in the PERMANOVA main tests were evaluated in terms of significance [48]. When the main test revealed statistical differences (p < 0.05), Permutation *t*-tests through PERMANOVA pairwise comparisons (PRIMER v6 with PERMANOVA+ add-on) were carried out amongst the different level of each significant factor. Coral cover data and field data group for mRNA expressions were tested for pairwise differences amongst the different levels of factor “depth” (i.e. 3 m vs 12 m for SA1, SA2, or TA). In the thermal stress data group, pairwise comparisons were performed: i) between T_0_ *vs* controls at the different time-points; ii) between thermal stress and controls at each time point. In any cases, the threshold of significance was set at p < 0.05.

The similarity matrix obtained from the field data group was also submitted to ordination analysis (performed by Principal Coordinate, PCO, analyses) and data clustering to assess whether coral nubbins sampled in different sites and at different depths can be discriminated by means of overall variations of mRNA profiles.

To generate heatmaps describing the overall transcriptional responses to temperature challenge, the thermal stress data group was submitted to data clustering using the Gene Cluster software ver 2.0 [49] and the TreeView software for cluster visualizations. Similarity was measured by standard correlation.

## Results

### *Pocillopora verrucosa* cover at the selected sampling sites in Bangka Island

**Fig 3:**
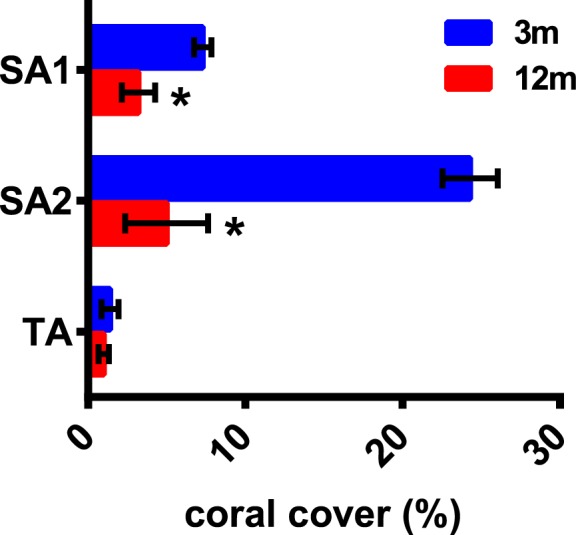
Percentage of cover assessed for *P*. *verrucosa* at the selected sampling sites and depths. Coral cover was estimated along 6 replicate 10 m line intercept transects at 3 and 12 m, considering the lowest low tide. Data are reported as mean ± s.d. (N = 6). *p < 0.05 12-m *vs* 3-m samples at each site (Permutation *t*-tests through PERMANOVA pairwise comparisons based on the Bray-Curtis resemblance matrix; 999 permutations).

Percentage of cover was evaluated as a proxy for habitat suitability conditions for *P*. *verrucosa* at the selected sampling sites and depths (Fig 3). PERMANOVA analyses indicated that the single factors “site” and “depth” significantly affected coral cover, and also that the two factors displayed significant interactive effects (p < 0.001; Table 3). Permutation *t*-tests showed that coral cover at 12 m was significantly reduced both at sites SA1 and SA2, whereas no significant depth-related differences were at the site TA, which showed the lowest percentage values at both depths (Fig 3).

**Table 3:** Results of PERMANOVA analyses on percentage of cover and transcript expressions of *P*. *verrucosa* under field conditions.

Source of Variation	df	Coral cover		mRNA expression data					
				*ATPs*		*hsp70*		*ndh*	
		Pseudo-F	P	Pseudo-F	P	Pseudo-F	P	Pseudo-F	P
**site**	2	**57.75**	**0.001**	**4.09**	**0.04**	2.05	0.159	7.21 x10^-2^	0.930
**depth**	1	**24.18**	**0.001**	3.96	0.07	**47.4**	**0.001**	**6.88**	**0.016**
**site x depth**	2	**5.65**	**0.001**	**5.75**	**0.01**	1.51	0.259	**4.69**	**0.029**

df = degree of freedom; Pseudo-F = F value by permutation [48]; P (perm): probability of pseudo-F.

### Transcript profiles in field sample group

**Fig 4:**
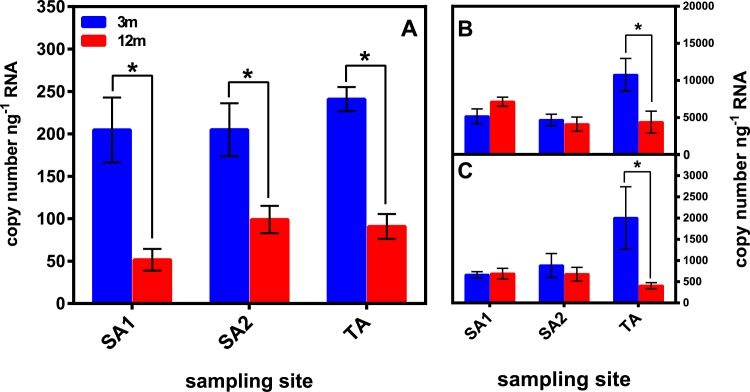
Expression profiles of a *hsp70* transcript in *P*. *verrucosa* nubbins collected at different sites and depths. *Hsp70* copy number variations **(A)**. Transcript levels of an ATP synthase (*ATPs*) **(B**) and of a NADH dehydrogenase (*ndh*) **(C)**. Data are expressed as mean ± s.e.m. (N = 6) of the copy numbers of each gene product normalized over the nanograms of total RNA employed in a single qPCR reaction. *p < 0.05 12-m *vs* 3-m samples within each site (Permutation *t*-tests through PERMANOVA pairwise comparisons using Euclidean Distance resemblance matrix; 999 permutations).

Copy number variations of the *hsp70* gene product were assessed in *P*. *verrucosa* sampled at different sites and depths (Fig 4A). Results from PERMANOVA analyses pointed out that “depth” significantly affected *hsp70* expression, and no significant interaction between the factors “depth” and “sampling site” was observed (Table 3). Permutation *t*-tests showed that *hsp70* levels were significantly higher in 3-m than in 12-m collected corals at all sampling sites (Fig 4A).

Levels of metabolism-related transcripts *ATPs* and *ndh* were also assessed (Fig 4B and 4C). The single factors “sampling site” and “depth” significantly affected *ATPs* and *ndh* expressions, respectively, and a significant interaction between the two factors was observed for both transcripts (p< 0.05; Table 3). *ATPs* and *ndh* transcript levels showed significant differences between 3-m and 12-m collected corals only at the site TA (Fig 4B and 4C).

**Fig 5:**
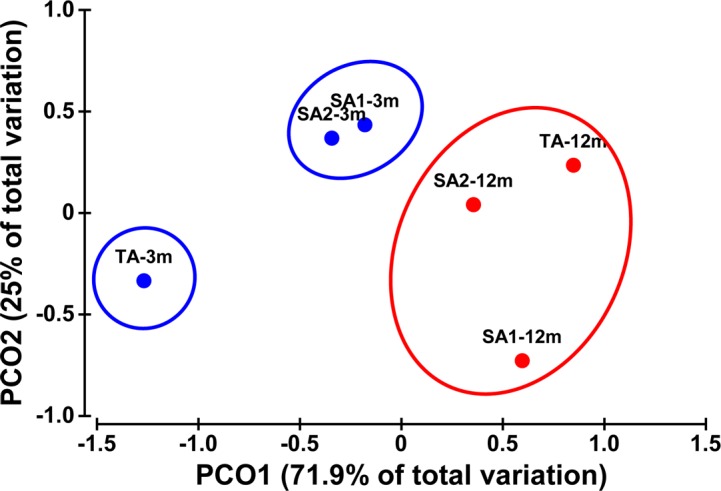
Principal coordinates ordination (PCO) bi-plot of whole mRNA expression levels (blu circles: samples collected at 3 m; red circles: samples collected at 12 m) with super-imposed cluster analysis (black lines) by condition (Euclidean Distance resemblance matrix; 999 permutations). SA1-3m: samples from SA1 collected at 3 m; SA1-12m: samples from SA1 collected at 12 m; SA2-3m: samples from SA2 collected at 3 m; SA2-12m: samples from SA2 collected at 12 m; TA-3m: samples from TA collected at 3 m; TA-12m: samples from TA collected at 12 m.

A PCO analysis was further applied to determine whether factors “sampling site” and “depth” can discriminate samples by means of overall variations of mRNA expressions for the selected gene products (Fig 5). Two principal coordinates (PCO1 and PCO2) were observed to explain 96.9% of total variance (71.9% and 25%, respectively). The super-imposed data clustering showed that samples from SA1 and SA2 collected at 3 m formed one group with evident separation from samples TA-3m (Fig 5). For all sampling sites, corals sampled at 12 m showed separation from those collected at 3 m, displaying a less pronounced site-to-site separation (Fig 5).

### Transcript profiles in thermal stress sample group

Changes of *hsp70* expression levels were evaluated during a short-term *P*. *verrucosa* exposure to thermal stress, and in control samples maintained at a constant water temperature (Fig 2B). Values were normalized against mRNA expression levels at the onset of the experimental exposure to thermal stress, achieved after a 14-day acclimation period to constant laboratory conditions (S3 Fig). During both the acclimatization period and the thermal stress exposure we daily checked coral color intensity using the coloring method described by Siebeck et al. [50]. On average, during the acclimatization period both 3-m and 12-m collected corals retained the color intensity originally owned at the sampling site/depth (all nubbins were scored between color level 5 to 6 in the color chart from Siebeck et al. [50]). Thermal stress decreased color intensity, with color loss being markedly more evident in 12-m collected samples (about 75% of nubbins were scored at the color level 2) compared to the 3-m samples (about 25% of nubbins scored at the color level 3) at all sites and after 7 days of thermal stress exposure.

**Fig 6:**
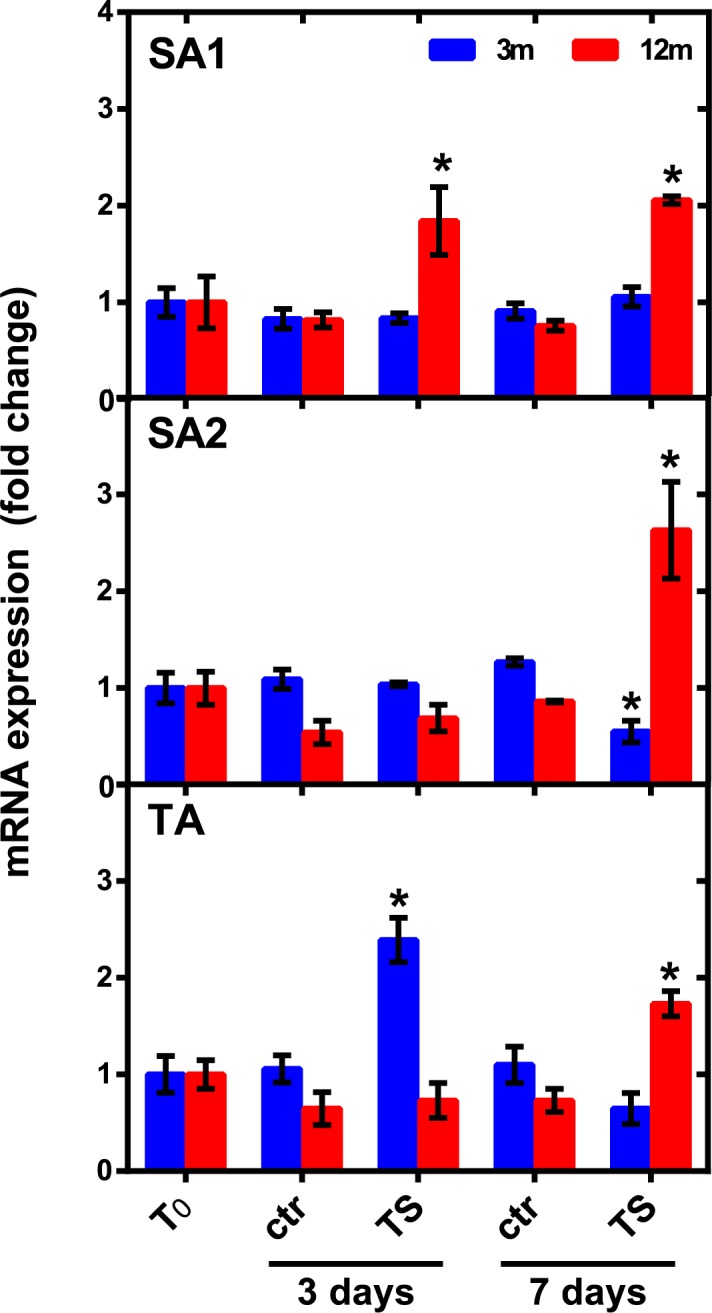
Effect of thermal stress on *hsp70* expression in *P*. *verrucosa* collected at different sites and depths. Fold changes were calculated with respect to *hsp70* mRNA levels assessed in nubbins after the 14-day acclimation period (T_0_). Values are expressed as mean ± s.e.m. (N = 6). Ctr: control samples; TS: samples subjected to thermal stress under the “nocturnal recovery” exposure scheme described in Fig 2. ^$^p < 0.5 Ctr *vs* T_0_, *p < 0.05 TS *vs* Ctr at respective time point (Permutation *t*-tests through PERMANOVA pairwise comparisons using Euclidean Distance resemblance matrix; 999 permutations).

Permutation *t*-tests showed that *hsp70* expression was not affected by thermal stress in 3-m collected nubbins of the SA1 and SA2 sites at any exposure time, although a significant down-regulation was observed at the SA2 site after 7 days of exposure (Fig 6). Nubbins collected at the TA site at 3 m showed significantly increased *hsp70* levels at 3 days compared with their respective controls (Fig 6). Coral nubbins collected at 12 m showed significantly increased *hsp70* levels starting from 3 days (SA1), or after 7 days (SA2 and TA) of exposure to thermal stress (Fig 6). PERMANOVA analyses are reported in S2 Table.

**Fig 7:**
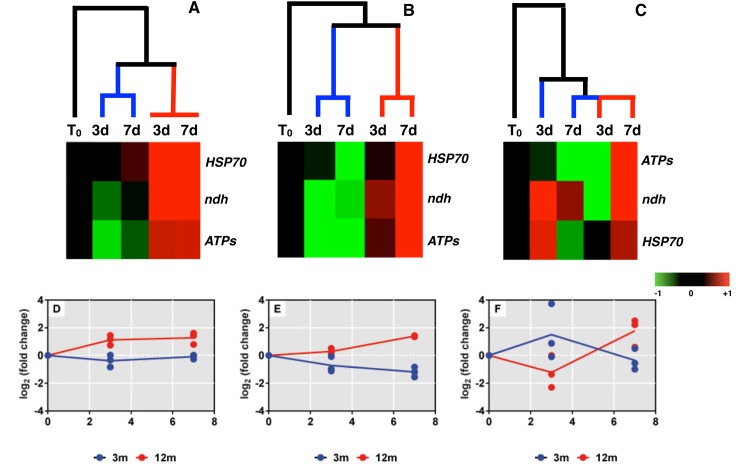
Comparisons of *P*. *verrucosa* mRNA expression responses to thermal stress among different sampling sites and depths. **(A,B,C)** Treatment ranking according to the qPCR data describing the transcriptional responses to increased temperature in coral nubbins collected at 3 m or 12 m. Hierarchical clustering was performed using fold change variations (log_2_-transformed) of *ATPs*, *ndh* and *hsp70* mRNA levels between thermally challenged nubbins and controls at each time point. Fold changes at each time-point were finally expressed as variations compared to levels assessed in nubbins after the 14-day acclimation period and before the onset of the thermal stress (T_0_), which served as the reference condition. Colors represent relative expression levels with respect to control corals at each time point. **(D,E,F)** Overview of the mRNA expression profiles (means) showing the transcriptional response to thermal stress in 3-m and 12-m collected corals. Dots represent point data for each gene product at the selected time-point, while solid lines represent the average trend of variations. Detailed data for transcript expression changes and related statistics are reported in S3 Fig (mRNA levels at the onset of the thermal stress exposure), Fig 6 (*hsp70* expression following thermal stress), and S4 Fig (*ATPs* and *ndh* expressions following thermal stress).

To help comparing overall transcriptional responses among datasets, fold change variations (log_2_-transformed) of *hsp70* expressions and those of *ATPs* and *ndh* were subjected to a cluster analysis (Fig 7). In-detail results of *ATPs* and *ndh* expression changes after the thermal stress exposure are reported in S4 Fig. Data clustering for sites SA1 and SA2 showed substantial time-course variations of the analysed transcript expressions, with samples from 3-m and 12-m collected corals forming distinct clusters (Fig 7). Corals collected at 3 m showed unchanged (SA1) or down-regulated (SA2) overall expression profiles, while corals collected at 12 m showed overall mRNA over-expressions after 3 days (SA1) and 7 days (SA1 and SA2) of exposure (Fig 7). No distinct clusters according to the depth of coral collection were observed at the site TA (Fig 7). A complex transcriptional response to thermal stress was displayed by corals collected at 3 m (Fig 7), particularly due to the *ndh* mRNA levels, which resulted at least 8-fold over-expressed with respect to the relative control at the 3-day time point, and returned to levels close to T_0_ at 7 days (S4 Fig). Corals collected at 12 m showed a time-course up-regulation of the selected transcripts similar to those observed at the SA1 and SA2 sampling sites (Fig 7).

## Discussion

Recent studies inferring physiological performances of *P*. *verrucosa* along 12° latitudes in the Red Sea showed an extensive phenotypic plasticity of the species despite the large geographic distances considered and the strong environmental gradients [51,52]. By contrast *P*. *verrucosa* showed a limited heterotrophic plasticity (i.e. the capacity of corals to acquire nutrients via zooplankton predation and the uptake of dissolved organic matter; [53]), and acclimation in regard to depth, which narrows its vertical distribution to shallow high-light environments [54,55]. To the best of our knowledge, this study investigated for the first time transcriptional stress responses in *P*. *verrucosa* that may underpin such physiological outputs, focusing on expression changes of a 70-kDa heat shock protein (Hsp70).

Given their key function as molecular chaperones regulating and preserving protein structure and functionality, Hsp70s are amongst those molecular mediators which mostly contribute to cellular defense and the physiological plasticity in marine organisms [56,57], and corals are no exceptions [45,58,59]. Regulation of *hsp70* expression was observed in coral response to thermal stress [42,59], ocean acidification [60], bacterial challenges [61], and environmental pollutants [62,63]. Amongst these multiple studies performed on different coral species, *hsp70* up-regulation in the initial stages of the response came out as a common pattern [59,64], leading to consider these transcripts as early-warning molecular markers of stress engaged to prevent the onset of pathological conditions [59]. Furthermore, since *hsp70s* are under diel cycle [65], their expression patterns may be affected by internal processes related to the regulation of the metabolic machinery. Although *Symbiodinium* diversity is believed to affect coral sensitivity/resilience to thermal stress and bleaching susceptibility, the molecular basis of coral responses to thermal stress associated with different *Symbiodinium* clades are not well understood [66,67]. A recent study pointed out that different putative species (genetically distinct types) as well as conspecific populations of *Symbiodinium* can confer differing levels of thermal tolerance to their coral host, but the genes that govern dinoflagellate thermal tolerance are unknown [67]. The influence of symbiosis on *P*. *verrucosa* transcriptional responses to thermal stress was not addressed in the present study. Nevertheless, this species has a well-documented stable host-symbiont partnership in response to environmental gradients [52,54,55,68]. It has been hypothesized that bleaching sensitivity of *P*. *verrucosa* may not be associated with *Symbiodinium* clade specificity, but rather it results from adaptive processes of the holobiont to local environmental conditions [68]. To account for stress effects on host cnidarian oxidative metabolism and regulation of symbiosis, this study also analysed expressions of ATP synthase (*ATPs*) and NADH dehydrogenase (*ndh*) transcripts [38,39]. Plasticity in metabolic rates is required to balance the energy budget and reallocate energy resources when facing a changed physiological status [39,69], and regulation of *ATPs* and *ndh* may occur in response to altered metabolic needs [59]. Furthermore, disruption of oxidative metabolism is a known initiation event in the activation of apoptotic and anti-apoptotic pathways [38]. The temporal dynamics of these processes may greatly influence coral sensitivity/recovery from stress [40,42,59].

As a general feature, members of the Hsp70 family may be functionally and structurally classified into two distinct groups: those that are constitutively expressed irrespective of the physiological status of the organism (Hsc70), and those that are stress-inducible (Hsp70), which are not expressed under normal conditions and whose transcription is promptly induced as the organism experiences adverse stressors [57]. The phylogenetic analysis reported in the Supplementary information pointed out that the *P*. *verrucosa hsp70* sequence employed in this study for the definition of qPCR primers and standards encodes a protein showing about 88% and 63% sequence homology with stress-inducible human and constitutively-expressed available scleractinian Hsp70s, respectively. This *hsp70* transcript was expressed at well detectable levels by all nubbins analysed both under basal conditions in the field and in controls (non-thermally stressed) from the laboratory experiments, in agreement with previous studies demonstrating that intertidal organisms display a minimal stress-inducible *hsp70* physiological expression to thrive more effectively with environmental variability [56,57].

The first stage of our experimental approach addressed the analysis of basal mRNA expression to set baseline physiological expression of the *hsp70* gene product in *P*. *verrucosa* and to unravel potential habitat influences. To this purpose, corals were sampled at two different depths from three sites, two (SA1 and SA2) representing coral banks located 400 m off the coast, one (TA) being an integral part of the coastal reef along the eastern side of the Bangka Island (North Sulawesi, Indonesia). Levels of *hsp70* expression in *P*. *verrucosa* nubbins followed a depth-related profile, with relatively higher expression levels being observed in 3-m collected nubbins compared to the 12-m ones regardless the sampling site investigated. These results are in agreement with previous studies reporting a higher *hsp70* physiological expression in coral populations experiencing thermally challenging habitats [15,30,42,45]. Furthermore, results from the PCO analysis, which comprised transcriptional profiles of *hsp70* and those of the *ATPs* and *ndh*, showed differences among sites when considering nubbins collected at 3 m. These differences resembled the relative geographical distances of the sites. Coral nubbins collected at 12 m from the different sites showed a less pronounced site-to-site separation. At such relatively higher depths environmental conditions may be more homogenous, even between more distant sites (i.e. between SA1/SA2 *vs* TA). On the whole, reported data suggested a different acclimatization response of *P*. *verrucosa* related to local habitat condition suitability which further agrees with the observed percentage of cover of the species between sites and depths. In particular, the TA site is likely to provide the less favorable habitat, which might explain the low percentage of cover (about 1%) at both depths. Compared with SA1 and SA2 sites, TA was characterized by relatively higher and less variable mean daily water temperatures (mean ± s.d.: 29°C ± 0.7°C at TA *vs* 27°C ± 1.2°C at SA1/SA2; S1 Fig). Other physical parameters did not show relevant differences. The possible influences of coastal hydrodinamism and further (unknown) stress factors may not be ruled out. The relatively higher *ndh* and *ATPs* expression levels observed in corals sampled at 3 m at the TA site suggested the occurrence of enhanced metabolic needs to satisfy the energetic demands for coping with these conditions [39,59,70]. However, *hsp70* levels in these samples were comparable with those of samples collected at the same depth at the SA1 and SA2 sites.

After acclimatization to a common and constant temperature in the laboratory, *P*. *verrucosa* nubbins were experimentally subjected to an altered thermal regime. According to Mayfield et al. [44], a nocturnal recovery experiment through a 7-days exposure period was performed, in which corals were exposed to elevated temperatures during the day (31°C, 3°C on average over the water temperature experienced in their natural environments) but mean ambient temperature (28°C) at night. This experimental approach may simulate a temperature profile that can characterize intertidal reefs at Bangka Island. Furthermore, given the peculiar transcriptional features of stress-inducible Hsp70s, which appear highly conserved between vertebrates and invertebrates (reviewed in Fabbri et al. [56] and Morris et al. [57]), it may prevent thermal accommodation likely occurring under a continuous heat stress exposure, as observed by Gates and Edmunds [71] in *Montastraea franksi*. A short-term exposure period (up to 7 days) was selected since inducible *hsp70s* are found to be engaged in the response to acute stresses and/or during the earlier phase of the stress response, after which further protective or compensative mechanisms maybe activated [28,57,59]. Therefore, changes of *hsp70* expression maybe lost or at least underestimated under prolonged exposures [21]. On the whole, this experimental setup may allow better analyzing the ability of *P*. *verrucosa* to promptly increase *hsp70* transcription within short-term heat stress exposures, as we previously reported for other intertidal species [27,28].

A different time-course response to thermal stress was observed between *P*. *verrucosa* nubbins collected at the different depths (regardless the sampling site), although acclimation to laboratory conditions for 14 days lead samples from the different field conditions to express almost similar *hsp70* levels. As previously hypothesized [3,72], data from basal *hsp70* expression analysis under field conditions may well explain such differential responses. Indeed, the lack of a *hsp70* response to heat stress in nubbins collected at 3 m may arise from the observed relatively higher physiological *hsp70* expression levels. As previously observed in other corals species [45] and, more generally, in different intertidal invertebrates [28,30], this may have provided a preparative defense strategy for protection against further stress conditions as that represented by the thermal stress employed in this study. The molecular basis of this expression pattern has been well explained in vertebrates, where high Hsp70 levels may act as a “molecular thermometer” inhibiting the synthesis of new Hsp70 molecules [73]. The different time-course of the *hsp70* expression showed by nubbins from the TA site may be due to the peculiar local habitat conditions faced by these samples in the environment, which may have prompt the TA-sampled nubbins to be more sensitive towards the thermal stress compared with the SA1 and SA2 samples. Levin et al. [67] recently demonstrated that *Symbiodinium* cultures isolated from thermo-sensitive coral populations suffered a significant decrease in photosynthetic efficiency and increase in ROS cell leakage when subjected to thermal stress (13 days at 32°C), whereas cultures from more thermo-tolerant populations showed no signs of physiological stress. The authors hypothesized that while the observed transcriptional response by the thermo-tolerant *Symbiodinium* may allow maintaining symbiosis with their coral host at elevated temperature, stress effects induced in thermo-sensitive *Symbiodinium* may cause oxidative damage to the coral host, resulting in bleaching. Given that *ATPs* and *ndh* are involved in maintenance of coral oxidative homeostasis, unchanged *ATPs* and *ndh* expression levels observed in thermally-challenged samples from sites SA1 and SA2 suggested that these nubbins may be overall more resilient towards a putative impairment of oxidative metabolism triggered by the applied thermal stress.

(Photo)autotrophy is the acquisition of carbon and nutrients from the photosynthetic byproducts of coral endosymbiotic algae. This process can provide up to 100% of a coral daily metabolic requirements to sustain physiological functions, including growth and calcification [74,75]. Therefore, when symbiont-derived carbon and nutrient sources are biased by a loss of algae photosynthetic efficiency, switching from an autotrophic to a heterotrophic nutrition (i.e. the capture of dissolved organic matter, particulate organic matter, and zooplankton [75]), may aid in maintaining coral holobiont fitness [76], because a particulate food supply compensates for the loss of autotrophic products. It also allows for the maintenance of higher symbiont and pigment concentrations within coral tissue, and thus facilitates the conservation of energy reserves [77]. A recent study on *Stylophora pistillata* reported the molecular pathways involved in the coral responses to light stress in relation to their nutritional status [53]. Well-fed heterotrophic corals better resisted the stress because feeding supplied antioxidants and energy-rich molecules that contributed to sustain protein- and DNA- repair mechanisms, thus protecting them from oxidative damages [53]. Unfed corals were affected by oxidative stress owing to a decrease in metabolic and energy processes, which ultimately lead to bleaching. Ziegler et al. [54] showed that *P*. *verrucosa* is photophysiologically well adapted to shallow high-light environments where it is most abundant, whereas it apparently lacks heterotrophic plasticity, as its percentage of cover significantly decreased below 10 m depths where autotrophy was reduced by decreased light levels. According to the findings described above, we may hypothesize that lack or reduced heterotrophic plasticity may contribute to enhance stress sensitivity of the species. Indeed, nubbins collected at 12 m appeared more sensitive to increased temperatures, since we observed a significant *hsp70* up-regulation that followed a time-dependent profile and was accompanied by a concomitant *ATPs* and *ndh* up-regulation, as a further signature for the developed stress condition due to thermal stress. Furthermore, it is worth noting that at 12 m corals may have experienced more stable and homogenous conditions in their natural environment, thus they retained relatively lower *hsp70* expression levels, which is likely to contribute to the enhanced stress sensitivity. This finding agrees with numerous studies showing that limits of thermotolerance in intertidal animals are related to their vertical zonation [30,78–80].

## Conclusion

This study showed that local habitat conditions have the potential to significantly influence transcription of stress-related genes *P*. *verrucosa*, further shaping its response capabilities to environment variability. Specifically, expression levels of a stress-inducible *hsp70* gene product in *P*. *verrucosa* resulted significantly influenced by vertical zonation, in agreement with the low capability for heterotrophic plasticity of the species [54], which is likely to decrease its stress tolerance. Furthermore, while in some species depth-dependent shifts of *Symbiodinium* types take place [81], several *Pocillopora* species displayed rather stable relationships with only a few *Symbiodinium* types, likely due to the vertical transmission of *Symbiodinium* in this genus [82]. This feature may be addressed as a hallmark of enhanced vulnerability of the species towards the consequences of environmental changes [54]. Nevertheless, the recent evidence provided by Levin et al. [67] is shading new light on the putative influence of the nature of symbiotic partnership in explaining coral response to stress. More extensive temporal studies on both coral host and *Symbiodinium* transcriptional responses would be necessary to identify the precise molecular events underlying depth-dependent *P*. *verrucosa* stress responses observed in this study.

Data on basal expression levels and heat stress effects in nubbins collected at relatively low depths agree with previous studies reporting extensive physiological plasticity of *P*. *verrucosa* with respect to temperature [51,83], and suggest that more challenging environments, within the limits of physiological windows [22], may boost protective responses providing enhanced resilience of the species towards environmental stressors.

## Supporting information

S1 Fig Satellite-derived physical parameter profiles at the selected sites across a 3- to 4- months period spanning the sampling time point. Mean daily values of temperature and salinity were retrieved using the Copernicus Marine Service Product GLOBAL_ANALYSIS_FORECAST_PHYS_001_002 (http://marine.copernicus.eu/), and visualized through the Panoply software ver 4.5 (http://www.giss.nasa.gov/tools/panoply/). Monthly average values of chlorophyll-a and photosynthetic active radiation (PAR) were retrieved through the GIOVANNI data system (MODIS-Aqua MODISA_L3m_CHL 4 km; MODIS-Aqua MODISA_L3m_FLH v2014 http://giovanni.gsfc.nasa.gov/giovanni/). SA1 and SA2 were considered as a unique SA site, given their close proximity. Inserts report box-and-whisker plots representing medians, upper and lower quartiles for each parameter. **p < 0.01 TA vs SA (Mann-Whitney U-test).
(PDF)

S2 Fig Phylogenetic relationships among Hsp70 deduced amino acid sequences of corals. (PDF)

S3 Fig Effects of acclimatization to the laboratory conditions. Expression profiles of a *hsp70* transcript was evaluated after a 14-days acclimatization period of *P*. *verrucosa* nubbins to the laboratory conditions (temperature 28°C; salinity 35 psu, light/dark cycle 10L:14D). *ATPs* and *ndh* mRNA levels in the same samples were also assessed to account for metabolic regulation. Values are expressed as mean ± s.e.m. (N = 6) of the copy numbers of each gene product normalized over the nanograms of total RNA employed in a single PCR reaction. SA1-3m: samples from SA1 collected at 3 m; SA1-12m: samples from SA1 collected at 12 m; SA2-3m: samples from SA2 collected at 3 m; SA2-12m: samples from SA2 collected at 12 m; TA-3m: samples from TA collected at 3 m; TA-12m: samples from TA collected at 12 m. *p < 0.05 according to permutation *t*-tests through PERMANOVA pairwise comparisons (Euclidean Distance resemblance matrix; 999 permutations).
(PDF)

S4 Fig In-detail expression profiles of *ATPs* and *ndh* transcripts in *P*. *verrucosa* following thermal stress exposure. Fold changes were calculated with respect to mRNA levels assessed in nubbins after the 14-day acclimation period (T_0_; S3 Fig). Values are expressed as mean ± s.e.m. (N = 6). Ctr: control samples; TS: samples subjected to thermal stress under the “nocturnal recovery” exposure described in Supplemental Information S1 File. ^$^p < 0.05 ctr *vs* T_0_ (Permutation *t*-tests through PERMANOVA pairwise comparisons based on Euclidean Distance resemblance matrix; 999 permutations); *p < 0.05 TS *vs* ctr at respective time point (Permutation *t*-tests through PERMANOVA pairwise comparisons based on Euclidean Distance resemblance matrix; 999 permutations).
(PDF)

S1 File Detailed description of the experimental setup for *P*. *verrucosa* thermal stress exposure. (DOCX)

S1 Table List of coral Hsp70 protein sequences employed for the phylogenetic analysis. (PDF)

S2 Table Results of PERMANOVA analyses on the effects of thermal stress on *P*. *verrucosa* nubbins transcript expressions. (PDF)
