# Supplementary figures and images for: Physiological plasticity related to zonation affects hsp70 expression in the reef-building coral Pocillopora verrucosa
Source: PLoS One. 2017 Feb 15;12(2):e0171456. doi: 10.1371/journal.pone.0171456 (PMC5310758; doi:10.1371/journal.pone.0171456)

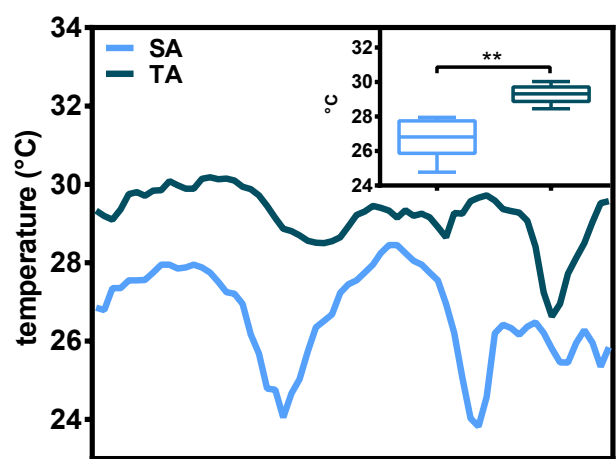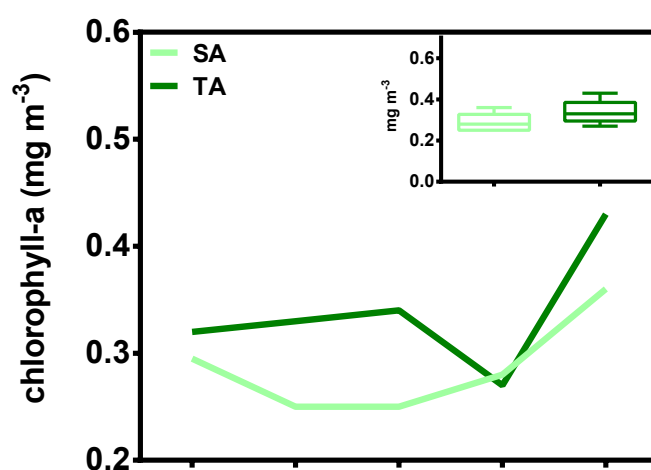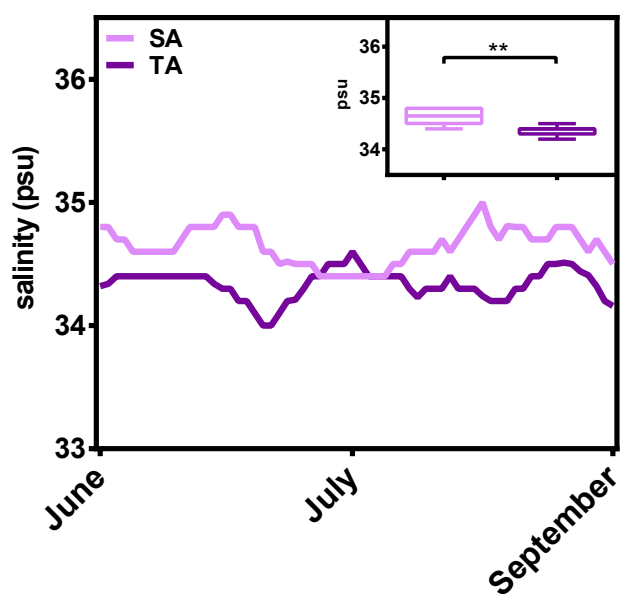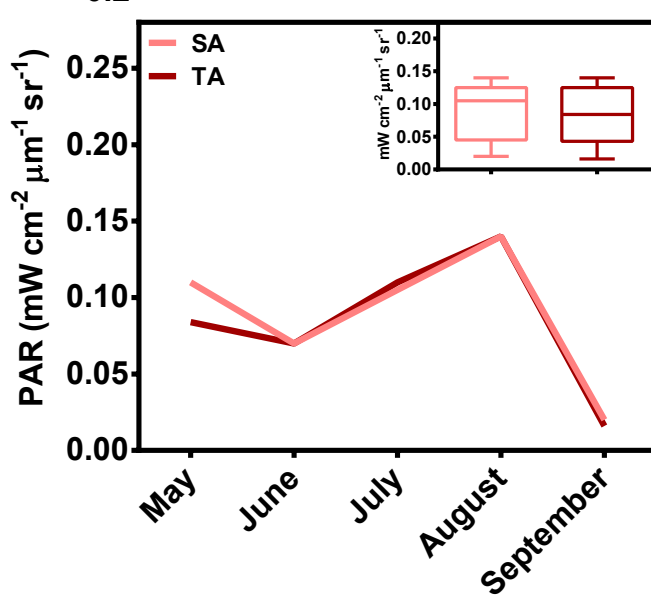

Supplement: S1 Fig — Mean daily values of temperature and salinity were retrieved using the Copernicus Marine Service Product GLOBAL_ANALYSIS_FORECAST_PHYS_001_002 (http://marine.copernicus.eu/), and visualized through the Panoply software ver 4.5 (http://www.giss.nasa.gov/tools/panoply/). Monthly average values of chlorophyll-a and photosynthetic active radiation (PAR) were retrieved through the GIOVANNI data system (MODIS-Aqua MODISA_L3m_CHL 4 km; MODIS-Aqua MODISA_L3m_FLH v2014 http://giovanni.gsfc.nasa.gov/giovanni/). SA1 and SA2 were considered as a unique SA site, given their close proximity. Inserts report box-and-whisker plots representing medians, upper and lower quartiles for each parameter. **p < 0.01 TA vs SA (Mann-Whitney U-test). (PDF) [file pone.0171456.s001.pdf]

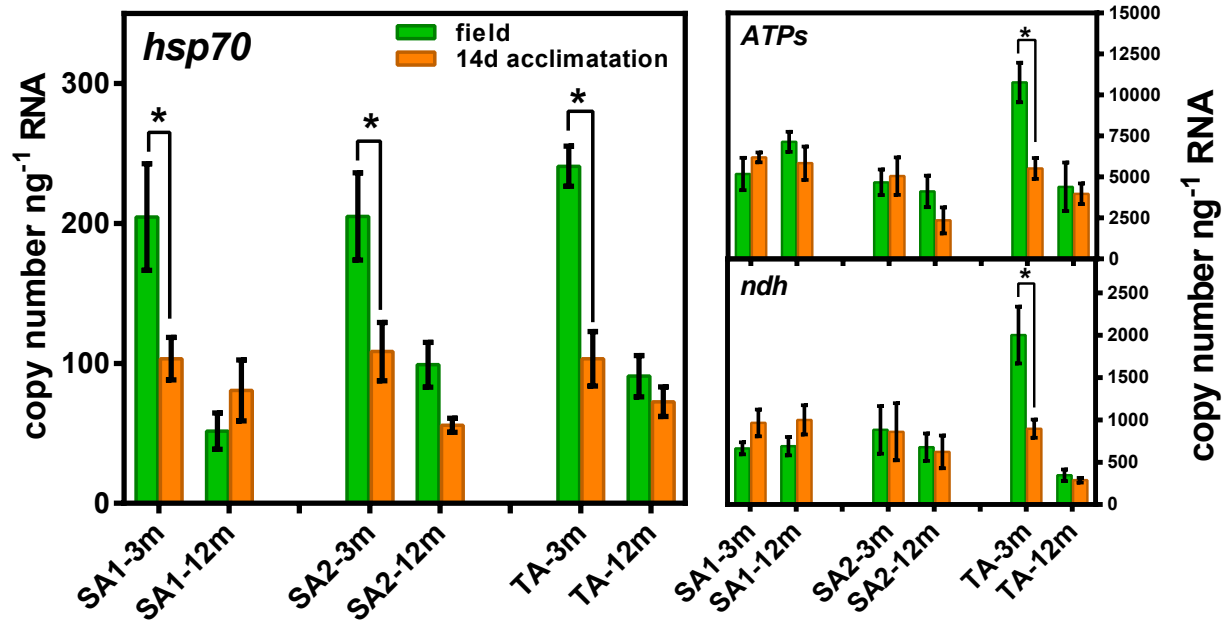

Supplement: S3 Fig — Expression profiles of a hsp70 transcript was evaluated after a 14-days acclimatization period of P. verrucosa nubbins to the laboratory conditions (temperature 28°C; salinity 35 psu, light/dark cycle 10L:14D). ATPs and ndh mRNA levels in the same samples were also assessed to account for metabolic regulation. Values are expressed as mean ± s.e.m. (N = 6) of the copy numbers of each gene product normalized over the nanograms of total RNA employed in a single PCR reaction. SA1-3m: samples from SA1 collected at 3 m; SA1-12m: samples from SA1 collected at 12 m; SA2-3m: samples from SA2 collected at 3 m; SA2-12m: samples from SA2 collected at 12 m; TA-3m: samples from TA collected at 3 m; TA-12m: samples from TA collected at 12 m. *p < 0.05 according to permutation t-tests through PERMANOVA pairwise comparisons (Euclidean Distance resemblance matrix; 999 permutations). (PDF) [file pone.0171456.s003.pdf]

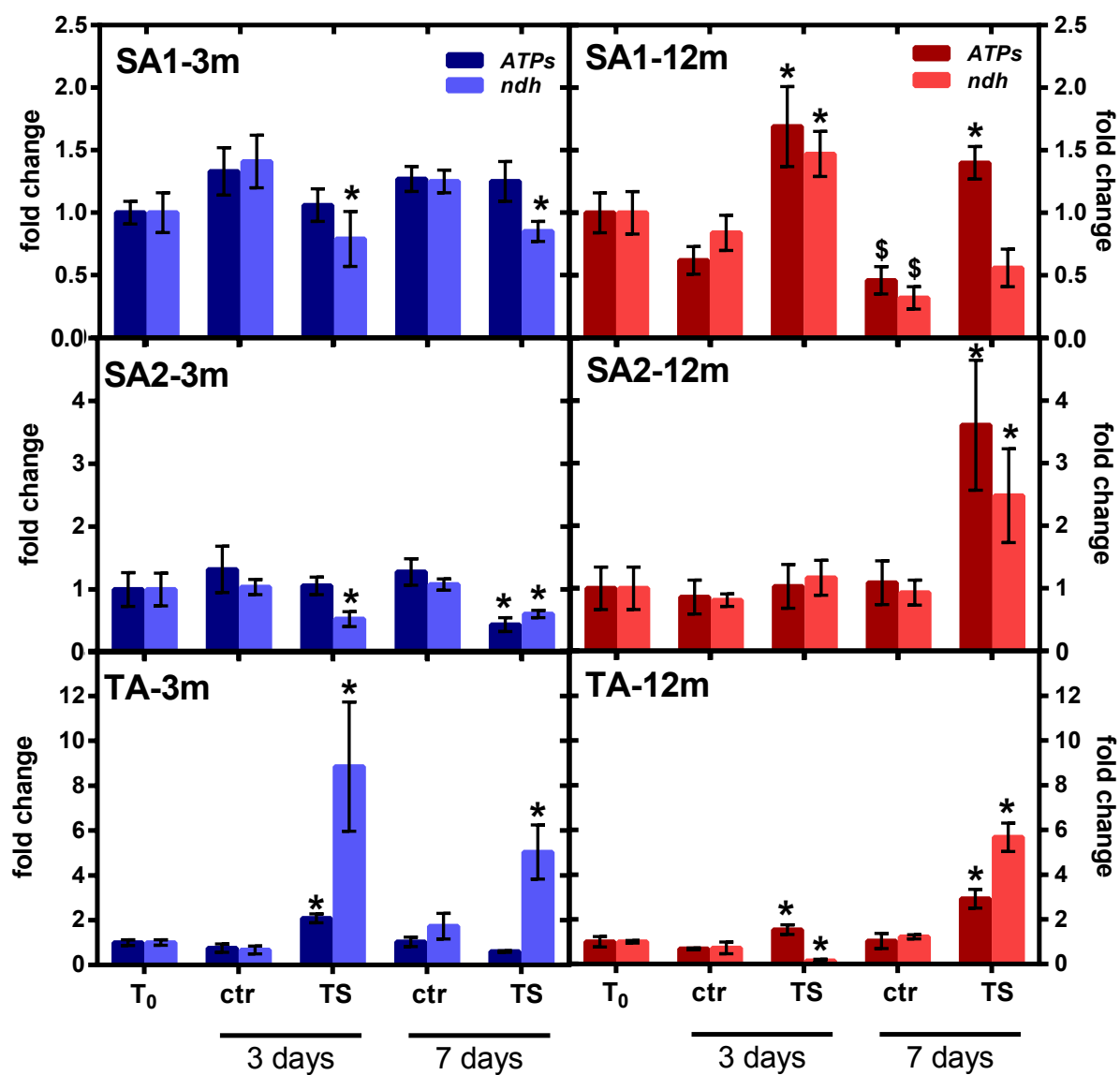

Supplement: S4 Fig — Fold changes were calculated with respect to mRNA levels assessed in nubbins after the 14-day acclimation period (T0; S3 Fig). Values are expressed as mean ± s.e.m. (N = 6). Ctr: control samples; TS: samples subjected to thermal stress under the “nocturnal recovery” exposure described in Supplemental Information S1 File. $p < 0.05 ctr vs T0 (Permutation t-tests through PERMANOVA pairwise comparisons based on Euclidean Distance resemblance matrix; 999 permutations); *p < 0.05 TS vs ctr at respective time point (Permutation t-tests through PERMANOVA pairwise comparisons based on Euclidean Distance resemblance matrix; 999 permutations). (PDF) [file pone.0171456.s004.pdf]
