# Supplementary material for: Physiological plasticity related to zonation affects hsp70 expression in the reef-building coral Pocillopora verrucosa
Source: PLoS One. 2017 Feb 15;12(2):e0171456. doi: 10.1371/journal.pone.0171456 (PMC5310758; doi:10.1371/journal.pone.0171456)
Supplement: S2 Fig — (PDF) [file pone.0171456.s002.pdf]

S2 Figure. Phylogenetic relationships among Hsp70 deduced amino acid sequences of corals. Multiple sequence alignment was generated with the MEGA software ver 6 [1] and the MUSCLE algorithm [2]. The tree was constructed by the Maximum Likelihood algorithm using PAUP* [3]. Bootstrap confidence values for the sequence groupings are indicated in the tree (N = 1000). The appropriate model parameters for the maximum-likelihood analysis were determined using a likelihood-ratio test with jModelTest [4]. The *P. verrucosa* Hsp70 sequence employed in this study is highlighted. The human Hsp70 (Human_HSP70, GenBank Ac. Numb. P17066) and Hsc70 (Human_HSC71, GenBank Ac. Numb. AAH19816) sequences were included in the alignment for comparison. SSA1 from *Saccharomyces cerevisae* was used as outgroup (GenBank Ac. Numb. P10591). Acronym explanations, accession numbers, and sequence features for coral protein sequences are reported in S1 Table.

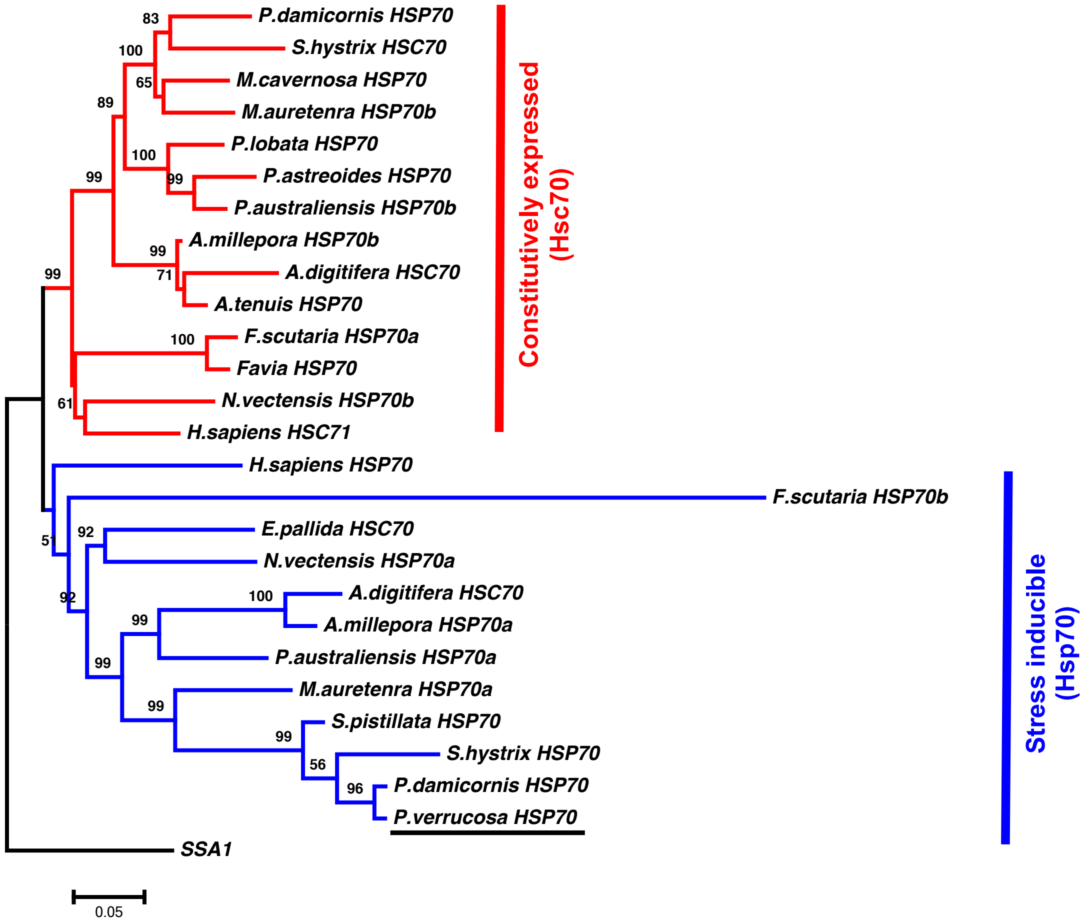

References

1. Tamura K, Stecher G, Peterson D, Filipski A, Kumar S. MEGA6: Molecular evolutionary genetics analysis version 6.0. Mol Biol Evol. 2013;30: 2725–2729. doi:10.1093/molbev/mst197
2. Edgar RC. MUSCLE: Multiple sequence alignment with high accuracy and high throughput. Nucleic Acids Res. 2004;32: 1792–1797. doi:10.1093/nar/gkh340
3. Swofford DL. PAUP*: phylogenetic analysis using parsimony, version 4.0b10. 21 Libr. 2003; 11pp. doi:citeulike-article-id:2345226
4. Posada D. jModelTest: Phylogenetic model averaging. Mol Biol Evol. 2008;25: 1253–1256. doi:10.1093/molbev/msn083
