# Supplementary material for: Physiological plasticity related to zonation affects hsp70 expression in the reef-building coral Pocillopora verrucosa
Source: PLoS One. 2017 Feb 15;12(2):e0171456. doi: 10.1371/journal.pone.0171456 (PMC5310758; doi:10.1371/journal.pone.0171456)
Supplement: S1 File — (DOCX) [file pone.0171456.s005.docx]

## Thermal stress exposure

For each sampling site, 6 colonies were collected at a 3-m and 12-m depth, respectively. The top of each colony was split into fragments of approximately 3 cm^2^ in size. One fragment for each colony was immediately stored in a suitable volume of the RNAlater® stabilization reagent (Sigma Aldrich, Milan, Italy) and maintained at -20°C to further assess transcriptional levels under field conditions (field sample group, N = 6). By fragmenting a single colony, we established replicates to account for sources of biological variability due to different sizes, shapes and thermal/light life histories of corals belonging to different colonies [1]. For the thermal stress sample group, we established independent replicates within each treatment group by using 6 aquaria for controls and thermal stress exposure, respectively, and considering this as the operative replication level (N = 6). The replication strategy is outlined in Fig I. Twelve fragments per colony, for a total 72 samples *per* site *per* depth, were randomly placed in the 12 aquaria filled with 20 L natural seawater each. A complete water change every 6 hours was performed gradually using a flow-through system which supplied water to the aquaria from 2 150-L tanks (each connected to 6 aquaria). Water parameters were monitored every 6 hours through a multi-parametric probe (ADWA AD12) and a densitometer (Milwaukee MR100 ATC). Each aquarium hosted one nubbin from each original colony per site per depth (FIG I). Corals spent a 14-days period of acclimatization at a constant water temperature of 28°C ± 0.5°C and a photoperiod of 10h:14h light:dark daily cycles resembling the average temperature and natural photoperiod at the collection sites (S1 Fig). The aquaria were equipped with 4 Boyu T8 lamps (white ligth bulbs: 40W, 1210mm; actinic blue light bulbs: 40W, 1210mm). Light levels in the aquaria were settled by performing an indirect evaluation of symbiont concentration inside the host tissue as a function of coral color intensity according to Siebeck et al. [2]. Based on the results of the tests performed during the field sampling procedures, light conditions in the aquaria were settled in order to maintain the same coloration owned at the original sampling site/depth.

Thermal stress experiment were carried out using a “nocturnal recovery” experimental profile [3]. After acclimatization, nubbins were collected from each treatment condition to account for animal physiological status at the onset of the experimental exposure to thermal stress, thus representing the “time zero” (T_0_) of the time-course evaluation (2 nubbins from two different colony/depth/site were pooled together to give one sample per aquaria; N = 6). The aquaria were randomly divided in two groups of controls, maintained at a constant temperature of 28°C ± 0.5°C, and thermal stress, in which water temperature was elevated at 31°C ± 0.5°C during the light phase, while reduced to 28°C ± 0.5°C during the dark phase, with a temperature ramp of 1°C hour^-1^. Temperature control was achieved both in the single aquaria and in the 150-L thanks, i.e. one thank and the connected 6 aquaria were settled for the control conditions, while the second tank and the connected 6 aquaria were settled for the thermal stress treatments.

Peak temperature for thermal stress was selected on the basis of previous studies in which a temperature increase of about 3°C over ambient temperature has been selected as a stress condition for corals [3,4]. For example, Barshis et al. [4] showed that a 3-days exposure to elevated temperature (3°C over ambient temperature) induced mortality in corals from moderately variable environments. A range of peak temperatures was also tested in preliminary experiments (from about 1°C to about 5°C over ambient temperature) to assure that the selected experimental setup established sub-lethal conditions and avoided coral death. Duration of temperature phases in the nocturnal recovery exposure scheme were chosen according to Mayfield et al. [3] to simulate a temperature profile that can characterize intertidal reefs at Bangka Island, and to prevent thermal accommodation likely occurring under a continuous heat stress exposure [5]. Temperature ramp was within the range reported by Kenkel et al. [6], which employed temperature ramps of 0.5 to 1.25 °C h^-1^ within a 5-hours exposure experiments. Bay et al. [7] employed a variable-temperature exposure scheme in which water temperatures fluctuated throughout the course of the day, mimicking tidal fluctuations, between 29 to 33°C in a 11-days exposure experiment. Although apparently rapid, these ramping rates are not unrealistic for natural coral populations [3,8–11].

Samplings from both the control and thermal-stress groups were performed at the same time of the day at which corals were collected from the field and after 3 and 7 days of treatment exposure. The short-term exposure timing was selected to account for acute effects of altered thermal conditions on *hsp70* transcription. Indeed, inducible hsp70s are found to be engaged in the response to acute stresses and/or during the earlier phase of the stress response, after which further protective or compensative mechanisms maybe activated [1,5,12]. Therefore, *hsp70* expression changes maybe lost or at least underestimated under prolonged exposures [12,13]. For each time point, 2 nubbins from two different colony/depth/site were pooled together to give one sample per aquaria (N = 6). Samples were immediately preserved in the RNAlater® solution (Sigma Aldrich, Milan, Italy) and stored at – 20°C until analysed.

**Fig I.** **Replication scheme employed in the thermal stress exposure experiments.** Detailed description is reported through the text. The different colors highlight the different sites. Hollow symbols indicate the 3-m collected samples; filled circles indicate the 12-m collected samples. Different hollow or filled symbols were employed to indicate that 6 different coral colonies were sampled for each site and depth.

**References**

1. Maor-Landaw K, Karako-Lampert S, Ben-Asher HW, Goffredo S, Falini G, Dubinsky Z, et al. Gene expression profiles during short-term heat stress in the red sea coral *Stylophora pistillata*. Glob Chang Biol. 2014; 1–10. doi:10.1111/gcb.12592

2. Siebeck UE, Marshall NJ, Klüter A, Hoegh-Guldberg O. Monitoring coral bleaching using a colour reference card. Coral Reefs. 2006;25: 453–460. doi:10.1007/s00338-006-0123-8

3. Mayfield AB, Chen M-N, Meng P-J, Lin H-J, Chen C-S, Liu P-J. The physiological response of the reef coral *Pocillopora damicornis* to elevated temperature: results from coral reef mesocosm experiments in Southern Taiwan. Mar Environ Res. 2013;86: 1–11. doi:10.1016/j.marenvres.2013.01.004

4. Barshis DJ, Ladner JT, Oliver TA, Seneca FO, Traylor-Knowles N, Palumbi SR. Genomic basis for coral resilience to climate change. Proc Natl Acad Sci. 2013;110: 1387–1392. doi:10.1073/pnas.1210224110

5. Morris JP, Thatje S, Hauton C. The use of stress-70 proteins in physiology: A re-appraisal. Mol Ecol. 2013;22: 1494–1502. doi:10.1111/mec.12216

6. Kenkel CD, Sheridan C, Leal MC, Bhagooli R, Castillo KD, Kurata N, et al. Diagnostic gene expression biomarkers of coral thermal stress. Mol Ecol Resour. 2014;14: 667–678. doi:10.1111/1755-0998.12218

7. Bay RA, Palumbi SR. Rapid acclimation ability mediated by transcriptome changes in reef-building corals. Genome Biol Evol. 2015;7: 1602–1612. doi:10.1093/gbe/evv085

8. Rosic NN, Pernice M, Dove S, Dunn S, Hoegh-Guldberg O. Gene expression profiles of cytosolic heat shock proteins Hsp70 and Hsp90 from symbiotic dinoflagellates in response to thermal stress: possible implications for coral bleaching. Cell Stress Chaperones. 2011;16: 69–80. doi:10.1007/s12192-010-0222-x

9. Berkelmans R. Time-integrated thermal bleaching thresholds of reefs and their variation on the Great Barrier Reef. Mar Ecol Prog Ser. 2002;229: 73–82. doi:10.3354/meps229073

10. Berkelmans R, Willis BL. Seasonal and local spatial patterns in the upper thermal limits of corals on the inshore Central Great Barrier Reef. Coral Reefs. 1999;18: 219–228. doi:10.1007/s003380050186

11. Dove S. Scleractinian corals with photoprotective host pigments are hypersensitive to thermal bleaching. Mar Ecol Prog Ser. 2004;272: 99–116. doi:10.3354/meps272099

12. Franzellitti S, Fabbri E. Differential HSP70 gene expression in the Mediterranean mussel exposed to various stressors. Biochem Biophys Res Commun. 2005;336: 1157–1163. doi:10.1016/j.bbrc.2005.08.244

13. Franzellitti S, Buratti S, Donnini F, Fabbri E. Exposure of mussels to a polluted environment: insights into the stress syndrome development. Comp Biochem Physiol Part C. 2010;152: 24–33. doi:10.1016/j.cbpc.2010.02.010
