# Supplementary material for: Physiological plasticity related to zonation affects hsp70 expression in the reef-building coral Pocillopora verrucosa
Source: PLoS One. 2017 Feb 15;12(2):e0171456. doi: 10.1371/journal.pone.0171456 (PMC5310758; doi:10.1371/journal.pone.0171456)
Supplement: S1 Table — (PDF) [file pone.0171456.s006.pdf]

S1 Table. List of coral Hsp70 protein sequences employed for the phylogenetic analysis

Species	Sequence name	Accession Number	Predicted functional classification[c]	Ref.
Acropora digitifera	**A.digitifera HSC70**	XP_015753975[a]	HSC70	[1]
	**A.digitifera HSC70**	XP_015748938[a]	HSC70*	[1]
Acropora millepora	**A.millepora HSP70a**	005613[b]	unclassified	[2]
	**A.millepora HSP70b**	c011524[b]	unclassified	[2]
Acropora tenuis	**A.tenuis HSP70**	6862[b]	unclassified putative HSC70	[2]
Exaiptasia pallida	**E.pallida HSC70**	KXJ28017[a]	HSC70	[3]
Favia sp.	**Favia HSP70**	50678[b]	unclassified putative HSC70	[2]
Fungia scutaria	**F.scutaria HSP70a**	51015[b]	unclassified putative HSC70	[2]
	**F. scutaria HSP70b**	3079[b]	unclassified putative HSP70	[2]
Madracis auretenra	**M.auretenra HSP70a**	34428[b]	unclassified putative HSP70	[2]
	**M. auretenra HSP70b**	39829[b]	unclassified putative HSC70	[2]
Montastraea cavernosa	**M.cavernosa HSP70**	74176[b]	unclassified putative HSC70	[2]
Nematostella vectensis	**N.vectensis HSP70a**	XP_001636593[a]	HSP70	[4]
	**N.vectensis HSP70b**	12168[b]	unclassified putative HSC70	[2]
Pocillopora damicornis	**P.damicornis HSP70**	AFW20547[a]	HSP70	[5]
	**P. damicornis HSP70**	3293[b]	unclassified putative HSC70	[2]
Pocillopora verrucosa	**P.verrucosa HSP70**	AFW20582[a]	HSP70	[5]
Porites astrodies	**P.astroides HSP70**	3374[b]	unclassified putative HSC70	[2]
Porites australiensis	**P.australiensis HSP70a**	25149[b]	unclassified putative HSP70	[2]
	**P.australiensis HSP70b**	51024[b]	unclassified putative HSC70	[2]
Porites lobata	**P.lobata HSP70**	413[b]	unclassified putative HSC70	[2]
Seriatopora hystrix	**S.hystrix HSC70**	ADM13667[a]	HSC70	[2]
	**S.hystrix HSP70**	91906[b]	unclassified Putative HSP70	[2]
Stylophora pistillata	**S.pistillata HSP70**	AKC91104[a]	HSP70	[2]

[a]Accession number for amino acid sequences retrieved in the NCBI GenBank (http://www.ncbi.nlm.nih.gov/genbank/).

[b]Identification number for amino acid sequences retrieved in the Reefgenomics database (http://comparative.reefgenomics.org; [2]). The database was interrogated using *A. digitifera* Hsp70 or Hsc70 as query sequences through the SequenceServer ver 1.0.2 BLAST tool (http://www.sequenceserver.com; [6]).

[c]Putative functional classification of unclassified sequences was based on BLAST analysis (http://blast.ncbi.nlm.nih.gov/Blast.cgi) and on multiple sequence alignment which assessed the occurrence of conserved Hsc70 or Hsp70 specific sequence signatures [7].
*70-90% homology with Hsp70 sequences; about 60% homology with Hsc70 sequences

References

1. Shinzato C, Shoguchi E, Kawashima T, Hamada M, Hisata K, Tanaka M, et al. Using the *Acropora digitifera* genome to understand coral responses to environmental change. Nature. Nature Publishing Group; 2011;476: 320–323. doi:10.1038/nature10249
2. Bhattacharya D, Agrawal S, Aranda M, Baumgarten S, Belcaid M, Drake JL, et al. Comparative genomics explains the evolutionary success of reef-forming corals. Elife. 2016;5: 1–26. doi:10.7554/eLife.13288
3. Baumgarten S, Simakov O, Esherick LY, Liew YJ, Lehnert EM, Michell CT, et al. The genome of *Aiptasia*, a sea anemone model for coral symbiosis. Proc Natl Acad Sci U S A. 2015;112: 11893–8. doi:10.1073/pnas.1513318112
4. Putnam NH, Srivastava M, Hellsten U, Dirks B, Chapman J, Salamov A, et al. Sea anemone genome reveals ancestral eumetazoan gene repertoire and genomic organization. Science. 2007;317: 86–94. doi:10.1126/science.1139158
5. Schmidt-Roach S, Lundgren P, Miller KJ, Gerlach G, Noreen AME, Andreakis N. Assessing hidden species diversity in the coral *Pocillopora damicornis* from Eastern Australia. Coral Reefs. 2013;32: 161–172. doi:10.1007/s00338-012-0959-z
6. Priyam A, Woodcroft BJ, Rai V, Munagala A, Moghul I, Ter F, et al. Sequenceserver: a modern graphical user interface for custom BLAST databases. bioRxiv. Cold Spring Harbor Labs Journals; 2015;33142. doi:10.1101/033142
7. Fabbri E, Valbonesi P, Franzellitti S. HSP expression in bivalves. Invertebr Surviv J. 2008;5: 135–161.
