# Supplementary material for: Physiological plasticity related to zonation affects hsp70 expression in the reef-building coral Pocillopora verrucosa
Source: PLoS One. 2017 Feb 15;12(2):e0171456. doi: 10.1371/journal.pone.0171456 (PMC5310758; doi:10.1371/journal.pone.0171456)
Supplement: S2 Table — (PDF) [file pone.0171456.s007.pdf]

S2 Table. Results of PERMANOVA analyses on the effects of thermal stress on *P. verrucosa* nubbins transcript expressions.

Source of variation	df	Whole dataset		*ATPs*		*ndh*		*hsp70*	
		Pseudo-F	P	Pseudo-F	P	Pseudo-F	P	Pseudo-F	P
**SI**	2	20.54	0.001	1.60	0.179	28.60	0.001	0.47	0.651
**DE**	1	4.70	0.006	3.21	0.064	5.39	0.005	3.12	0.038
**TI**	2	7.36	0.001	8.11	0.002	7.63	0.001	3.73	0.033
**TR**	1	29.33	0.001	18.65	0.001	31.93	0.001	32.83	0.001
**SIxDE**	2	7.51	0.001	5.76	0.004	7.73	0.001	9.62	0.001
**SIxTI**	4	5.95	0.001	1.72	0.149	7.46	0.001	3.62	0.014
**SIxTR**	2	19.51	0.001	1.97	0.147	26.93	0.001	1.34	0.279
**DExTI**	2	12.73	0.001	13.34	0.001	11.40	0.001	21.47	0.001
**DExTR**	1	8.47	0.001	27.23	0.001	0.74	0.409	26.37	0.001
**TIxTR**	2	7.55	0.001	5.58	0.006	7.99	0.001	8.47	0.001
**SIxDExTI**	4	11.89	0.001	6.10	0.001	14.10	0.001	7.73	0.001
**SIxDExTR**	2	9.89	0.001	1.29	0.262	13.00	0.001	4.89	0.012
**SIxTIxTR**	4	5.68	0.001	2.51	0.044	7.04	0.001	2.23	0.070
**DExTIxTR**	2	15.17	0.001	20.86	0.001	11.96	0.001	27.15	0.001
**SIxDExTIxTR**	4	10.87	0.001	3.72	0.006	13.57	0.001	5.89	0.001

SI: sampling site (SA1, SA2, TA); DE: depth (3 m, 12 m); TI: exposure time (0, 3, 7 days); TR: experimental treatment (control, termal stress exposure); df = degree of freedom; Pseudo-F = F value by permutation; P (perm): probability of pseudo-F.
